# Identification of a novel *DDB2* mutation in a Chinese Han family with Xeroderma pigmentosum group E:a case report and literature review

**DOI:** 10.1186/s12881-020-00997-0

**Published:** 2020-03-30

**Authors:** Rui Yang, Qingtao Kong, Yuanyuan Duan, Weiwei Li, Hong Sang

**Affiliations:** 1 Department of Dermatology, Jinling Hospital, Nanjing University, School of Medicine, Nanjing, 210002 China; 2 Dermatology, Chinese Academy of Medical Sciences and Peking Union Medical College, Nanjing, China; 3 Department of Reproduction and Genetics, Institute of Laboratory Medicine, Jinling Hospital, Nanjing University School of Medicine, Nanjing, 210002 China

**Keywords:** Xeroderma pigmentosum, *DDB2* gene, Novel mutation, Skin cancer, Case report

## Abstract

**Background:**

Xeroderma pigmentosum (XP) is a rare autosomal recessive genodermatosis. There are eight complementation groups of XP (XP-A to G and a variant form). XP-E is one of the least common forms, and XP-E patients are generally not diagnosed until they are adults due to a later onset of skin alterations.

**Case presentation:**

We report a case of a 28-year-old Chinese woman with freckle-like hyperpigmented macules in a sun-exposed area who is prone to develop basal cell carcinomas. A genetic study revealed a novel homozygous c.111_112del deletion in exon 1 of the *DDB2* gene. Western blotting analysis revealed that the patient lacked the expression of the wild-type mature DDB2 protein. The proband was first diagnosed with XPE on the basis of clinical findings and genetic testing. Sun protection was recommended, and the patient did not develop any skin cancers during the one-year follow-up.

**Conclusions:**

We identified a novel homozygous deletion in *DDB2* gene in Chinese XP-E patients having unique clinical features.

## Background

Xeroderma pigmentosum (XP) is a rare autosomal recessive genetic disease characterized by increased sensitivity to ultraviolet radiation-induced sunburn, skin pigmentation, skin cancers, ocular disease and neurological degeneration. XP is classified into eight genetic groups: groups A-G and a variant form (XP-V). The genes responsible for XP-A to XP-G and XP-V are *XPA, ERCC3, XPC, ERCC2, DDB2, ERCC4, ERCC5,* and *POLH* [1]. It is estimated that the prevalence of XP is 1/1,000,000 in the USA and Europe, and it is more common in Japan, with an estimated of 1/22,000 [2, 3]. Based on the different mutations of the XP gene, there are at least six different clinical symptoms: XP, XP with neurological symptoms, trichothiodystrophy, XP with trichothiodystrophy symptoms, XP with Cockayne syndrome and cerebro-oculo-facio-skeletal syndrome [4].

Severe sunburn on minimal sun exposure is a conspicuous symptom for the diagnosis of XP, but only approximately 60% of all patients show this manifestation [5]. XP is primarily diagnosed in the clinic and confirmed by functional cell-based systems as well as genetic tests. Because of a deficiency in the restoration of DNA damage induced by UV radiation, patients with XP have a 2000-fold increased risk of melanoma and a 10,000-fold increased risk of nonmelanoma skin cancer [6]. Early diagnosis is of utmost importance to initiate strict and consistent sun protection and regular examination of the skin.

XP-E is one of the least common forms of XP. XP-E patients are generally not diagnosed until they are adults due to the lack of sunburn reaction, and they are prone to develop multiple skin cancers, such as basal cell carcinoma, squamous cell carcinoma and malignant melanoma. DNA-binding protein is a heterodimer of DDB1 and DDB2 and is very important for proper DNA damage recognition involved in nucleotide excision repair (NER) [7]. Mutations of the *DDB2* gene are responsible for XP-E.

Here, we report an XP-E patient from a Chinese consanguineous family. The patient harbours a novel homozygous mutation, c.111_112del (p.A39Efs*6), of the *DDB2* gene. To our knowledge, XP-E has not yet been reported in Chinese people.

## Case presentation

**Fig. 1:**
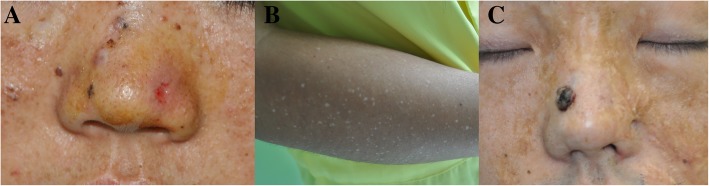
Clinical findings of the proband and her brother. **a**. Freckle-like hyperpigmented macules on her face with an ulcerated bleeding nodule on her nose. **b**. Hyper- and hypopigmented macules on her arm. **c**. The proband’s brother has similar clinical characteristics

One year ago, a 28-year-old Chinese woman came to our department to consult for recurrent ulceration, bleeding and crusted nodules on her nose. She is the first child of healthy consanguineous parents. The parents of this patient were first cousins. Pregnancy and delivery were normal. She has normal mental development. On examination, numerous freckle-like hyperpigmented macules were seen on her face and neck with an ulcerated bleeding nodule on her nose (Fig. 1a). Reticulate hyper- and hypopigmented macules could be seen on her arm (Fig. 1b). The skin throughout her body was dry and could not be relieved by using cream. She denied history of acute sunburn. Her brother, who suffered burns 5 years ago, also had similar clinical characteristics to the proband. An extensive burn scar and a 0.5 cm × 1 cm hyperpigmented nodule were seen on the face of her brother (Fig. 1c). Biopsy results confirmed the diagnosis of basal cell carcinomas of the hyperpigmented nodules on the nose of the proband and her brother.

### Genetic study

**Fig. 2:**
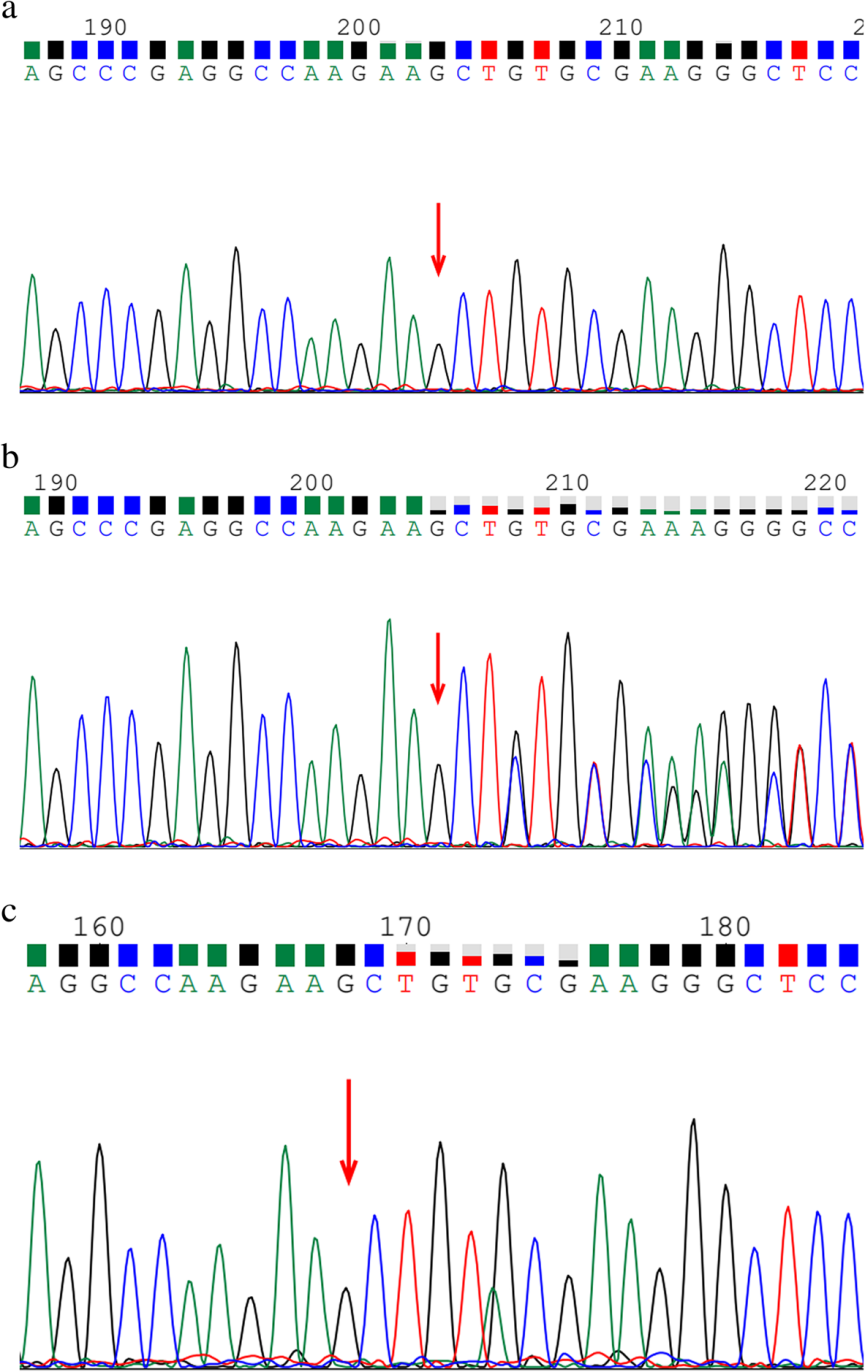
Novel deletion mutation in the DDB2 gene was detected in the patient. **a**. Homozygous mutation. **b**. Heterozygous state. **c**. Confirmation of the mutation at the cDNA level

**Fig. 3:**
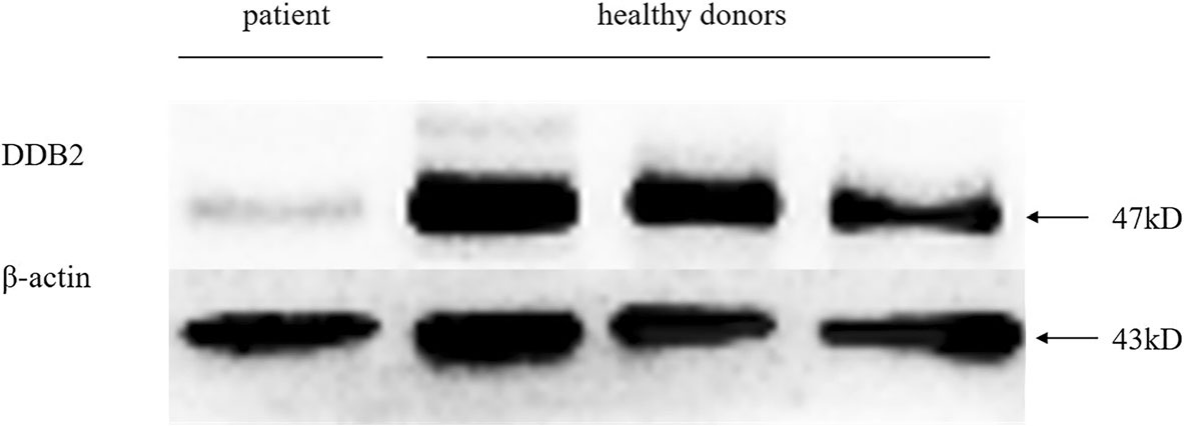
Western blotting analysis revealed that the patient lacked the expression of the wild-type mature DDB2 protein

This study was approved by the Ethics Committee of Jinling Hospital and written informed consent was obtained from all participants. Blood samples of the patient and her family members were collected. DNAs were isolated from blood samples. Total mRNA was extracted from the proband’s surgical tissue. cDNA was obtained by reverse transcription. Eight genes responsible for XP were sequenced using next-generation sequencing (NGS), and the result was confirmed by Sanger sequencing. A novel homozygous c.111_112del deletion in exon 1 of the *DDB2* gene, which resulted in a frameshift mutation of amino acid p.A39Efs*6, was detected in the proband and her brother (Fig. 2a). Her parents are heterozygous for this mutation (Fig. 2b). Sequencing at the cDNA level also confirmed the mutation (Fig. 2c). This mutation was not found in 120 unrelated, population-matched control individuals. Western blotting analysis revealed that the patient lacked the expression of the wild-type mature DDB2 protein (Fig. 3). The proband and her brother were diagnosed with XPE on the basis of clinical findings and genetic tests. Sun protection was recommended, and the patient did not develop any skin cancers within 1 year of follow-up.

## Discussion and conclusions

Three pathways in mammalian cells are involved in protection from UV irradiation: global genomic repair, transcription-coupled repair and translesion synthesis. Nucleotide excision repair (NER), which includes both global genome repair and trans-coupled repair, can recognize and excise bulky helix-distorting lesions. Deficiencies in the NER pathway lead to several genetic disorders related to DNA repair, such as XP, trichothiodystrophy (TTD) and Cockayne syndrome (CS) [8].

Xeroderma pigmentosum (XP) is a rare autosomal recessive disorder that is genetically heterogeneous due to seven complementation groups with defective NER and a variant group (XP-V) with normal NER [9]. Xeroderma pigmentosum complementation group E (XP-E) is one of the least common groups of XP. Mutations in the *DDB2* gene are responsible for XP-E. DDB2 protein binds to DDB1 to form the UV-DDB complex, which recognizes UV-induced DNA damage and recruits proteins of the global repair pathway to initiate DNA repair [10]. The study of human primary cell lines from patients with XP-E containing *DDB2* mutations revealed that DDB2 was proposed to regulate the p53-mediated apoptotic pathway after UV irradiation [11]. XP-E patients usually have a normal sunburn response, but they have an increased frequency of skin cancer, such as basal cell carcinomas, squamous cell carcinoma and melanomas. Sunlight protection from an early age is of significant importance to prevent skin cancer in XP-E patients.

To the best of our knowledge, only 17 XP-E patients have been reported until now [12–14]. The mutations of *DDB2* identified in all XPE patients are summarized in Table 1. Substitution mutations (eleven of seventeen) were the most frequent mutation type. Mutations occur most frequently in exon 7 (five of eighteen) of *DDB2*. The frequency of XP-E is higher in white patients. Our patient is the only Chinese patient with XPE. Compared with other patients, our patients had fewer tumours and better prognosis within the one-year follow-up. The reason for the phenotypic variations in XPE patients may depend on climate and lifestyle.

**Table 1:** Summary of *DDB2* mutations based on GenBank accession no. NM_000107.3

Patient	Ethnicity	Nucleotide	Protein	Exon	Reference
A 28-year-old woman	Chinese	c.111_112del	p.A39Efs*6	1	This paper
XP23V	Italian	c.703_1023del	p.Leu235_Lys341del	4,5,6	Rapic-Otrin, et al. Hum Mol Genet. 2003 Jul 1;12(13):1507–22.
XP27V	Italian	c.730_733delAAAG	p.Lys244X	4	
		c.703_880del	p.Trp236Valfs*	4,5	
		c.703_1023del	p.Leu235_Lys341del	4,5,6	
XP25V	Italian	c.919G > T	p.Asp307Tyr	6	
GM01389	American	c.1049 T > C	p.Leu350Pro	7	
		c.1045_1047delAAC	p.Asn349del	7	
XP98BR	Caucasian	c.161G > A	p.Trp54X	1	Fassihi, et al. Proc Natl Acad Sci U S A. 2016 Mar 1;113(9):E1236–45
XP105BR	Caucasian	c.1070C > T	p.Pro357Leu	7	
		c.716G > T	p.Arg239Ile	4	
XP100BR	Caucasian	c.457-2A > C	Splice	3	
XP115BR	Pakistani	c.1149delG	p.Met383fs	7	
XP82TO	Japanese	c.730A > G	p.Lys244Glu	4	Nichols, et al. J Biol Chem. 1996 Oct 4;271(40):24317–20.
XP2RO	Dutch	c.818G > A	p.Arg273His	5	
XP51MAN-1	Tunisian	c.1138delG	p.Lys381Argfs*2	7	Ben Rekaya, et al. J Dermatol Sci. 2018 Feb;89(2):172–180.
A 12-year-old boy	unknown	c.574C > T	p.Arg192X	3	Vahteristo, et al. J Med Genet. 2007 Nov;44(11):718–20.
Ops1	Japanese	c.937C > T	p.Arg313X	6	Itoh, et al. J Invest Dermatol. 1999 Aug;113(2):251–7.
XP37BE	American/Dutch	c.818G > A	p.Arg273His	5	Oh, et al. J Invest Dermatol. 2011 Mar;131(3):785–8.
XP66BE					
XP1GO	German	c.914C > A	p.Thr305Asn	6	
XP408BE	American	c.1049 T > C	p.Leu350Pro	7	
		c.1045_1047delAAC	p.Asn349del	7	

In this study, we identified a novel homozygous mutation, c.111_112del (p.A39Efs*6), of the *DDB2* gene in an adult XP-E patient from a Chinese consanguineous family and summarized all mutations of *DDB2*. This is the first report of XP-E in Chinese people. After an exact diagnosis, strict sun protection was suggested, and the patients received a benefit from this management.

## Data Availability

The datasets generated and/or analysed during the current study are available in the [https://pan.baidu.com/] repository, https://pan.baidu.com/s/1h6PBB2QV_DGHYLUmF-ztow , access code: tai6. Usename: yrforbmc; password: yangrui417.

## Abbreviations

XP: Xeroderma pigmentosum
XPE: Xeroderma pigmentosum group E
